# Nonretinocentric localization of successively presented flashes during smooth pursuit eye movements

**DOI:** 10.1167/jov.20.4.8

**Published:** 2020-04-16

**Authors:** Stefan Dowiasch, Sonia Meyer-Stender, Steffen Klingenhoefer, Frank Bremmer

**Affiliations:** Department of Neurophysics, University of Marburg, Marburg, Germany; Center for Mind, Brain and Behavior (CMBB), University of Marburg and Justus-Liebig-University Giessen, Marburg, Germany; Thomas RECORDING GmbH, Giessen, Germany; Department of Neurophysics, University of Marburg, Marburg, Germany; Department of Neurophysics, University of Marburg, Marburg, Germany; Center for Molecular and Behavioral Neuroscience, Rutgers University, Newark, NJ, USA; Department of Neurophysics, University of Marburg, Marburg, Germany; Center for Mind, Brain and Behavior (CMBB), University of Marburg and Justus-Liebig-University Giessen, Marburg, Germany

**Keywords:** localization error, perception, smooth pursuit, reference frame, successive flashes

## Abstract

Keeping track of objects in our environment across body and eye movements is essential for perceptual stability and localization of external objects. As of yet, it is largely unknown how this perceptual stability is achieved. A common behavioral approach to investigate potential neuronal mechanisms underlying spatial vision has been the presentation of one brief visual stimulus across eye movements. Here, we adopted this approach and aimed to determine the reference frame of the perceptual localization of two successively presented flashes during fixation and smooth pursuit eye movements (SPEMs). To this end, eccentric flashes with a stimulus onset asynchrony of zero or ± 200 ms had to be localized with respect to each other during fixation and SPEMs. The results were used to evaluate different models predicting the reference frame in which the spatial information is represented. First, we were able to reproduce the well-known effect of relative mislocalization during fixation. Second, smooth pursuit led to a characteristic relative mislocalization, different from that during fixation. A model assuming that relative localization takes place in a nonretinocentric reference frame described our data best. This suggests that the relative localization judgment is performed at a stage of visual processing in which retinal and nonretinal information is available.

## Introduction

When navigating through our environment, we constantly move our eyes to redirect our gaze or to track objects of interest in order to approach targets or to avoid obstacles. Despite this constant movement of our eyes and bodies, we perceive the world around us as stable. In order to obtain such a stable percept of the outside world, it has been hypothesized that retinal (eye-centered) input signals are transformed to world-centered representations (Zipser & Andersen, 1988; Bremmer et al., 1998). Indeed, neurophysiological recordings in dorsal areas of nonhuman primates (NHPs) showed visual information being processed in different types of reference frames. Especially the multimodal ventral intraparietal (VIP) area, for which a functional equivalent has been found in humans (Bremmer et al., 2001), contains neurons encoding spatial information in eye-centered, head-centered, body-centered, world-centered, and intermediate reference frames (Duhamel et al., 1997; Avillac et al., 2005; Schlack et al., 2005; Chen et al., 2018).

According to this coordinate transformation hypothesis, an essential step in the course of the visual processing would be the transformation from a retinocentric (eye-centered) into a craniocentric (head-centered) frame of reference (Boussaoud & Bremmer, 1999; Salinas & Abbott, 2001). To this end, the visual system needs to combine information about the retinal location of a stimulus and information about the current eye position. For the latter, neural activity related to eye position (also known as “gain field” or “eye position field”) has been found in many areas of NHPs, for example, V1 (Trotter & Celebrini, 1999; Morris & Krekelberg, 2019), V4 (Bremmer, 2000), the middle temporal and the medial superior temporal area (Bremmer et al., 1997), and the VIP and lateral intraparietal areas (Bremmer et al., 1999; Morris et al., 2012; Morris et al., 2013; Morris et al., 2016). Indeed, it has been shown that decoded eye positions in such areas are accurate and sufficiently fast across eye movements (saccades and pursuit) to represent the actual eye position and therefore provide viable information for a coordinate transformation of visual signals from an eye-centered to a head-centered frame of reference (Morris et al., 2013; Morris et al., 2016; Dowiasch et al., 2016).

Yet, the visual system is only known to be very precise in determining the position of targets that are presented both with high contrast and long enough to be easily fixated, while systematical mislocalization effects occur when targets are presented briefly in the periphery or across eye movements (e.g., Adam et al., 1993; Bremmer & Krekelberg, 2003). In such situations, a mismatch between the actual eye position and its neuronal representation as well as distortions of the cortical spatial maps during eye movements may result in perceptual errors, i.e. objects being mislocalized in space. As an example, recent studies showed that eye position signals during eye movements are not always veridical. Instead, they show characteristic errors that could explain commonly found mislocalization effects of briefly flashed targets during saccades (Morris et al., 2012) and smooth pursuit eye movements (SPEMs) (Dowiasch et al., 2016).

During SPEMs, stationary targets are localized correctly, whereas flashed targets are mislocalized in the direction of the eye movement (Rotman et al., 2005). However, this mislocalization is not symmetric but significantly stronger ahead of the pursuit target and increases with more eccentric flash positions (van Beers et al., 2001; Königs & Bremmer, 2010). During fixation, flashed targets in the periphery are also misperceived, typically, closer toward the fovea (e.g., Mateeff & Gourevich, 1983; Kaminiarz et al., 2007; Königs & Bremmer, 2010). Yet, localization during fixation seems to depend on the exact experimental task, that is, whether an object is localized with respect to the body or with respect to a reference stimulus (e.g., Carrozzo et al., 2002; Eggert et al., 2001; Fortenbaugh et al., 2012; Kerzel, 2002). Müsseler and colleagues (1999) quantified this effect by investigating the relative localization of two eccentric stimuli briefly flashed during fixation. They found a significant bias toward less eccentric localization for the relative judgment of two flashes during fixation, when both targets were flashed with a stimulus onset asynchrony (SOA) of about 100 ms. However, this study could not differentiate if the target, the comparison stimulus, or both of them were actually mislocalized. Bocianski and colleagues (2008) extended this approach with different SOAs and showed that it was always the second stimulus that was perceived more centripetally with respect to the first. Furthermore, the relative localization depended on the stimulus onset asynchrony as well as the vertical distance between the two flashes.

With the current study, we aimed to further investigate localization of successively presented flashes by adding smooth pursuit eye movements to the original paradigm used by Bocianski et al. (2008). By comparing the relative localization performance of eccentrically flashed targets during fixation and SPEMs, we were able to distinguish between a shifting eye-centered or a head-/screen-centered reference frame. To this end, we developed different localization models, which either incorporated an additional shift of a retinocentric reference frame during SPEMs (e.g., by 2° for a SOA of 200 ms and a target speed of 10°/s) as compared to fixation or assumed no such difference. From comparing the model predictions with our behavioral data, we conclude that models assuming a relative localization judgment based on a nonretinocentric frame of reference described our results best. This might suggest that this localization process takes place at a later stage of visual processing, where the representation of both stimulus positions had already been transformed into a head- or even world-centered frame of reference.

## Materials and methods

### Subjects

Eight healthy human subjects (three male, five female; mean age: 25.6 ± 2.7 years) with normal or corrected-to-normal vision participated in the experiments. All of them had prior experience with behavioral experiments but were naive to the goals of the study. The procedures were approved by the local ethics committee and conformed to the Declaration of Helsinki. All participants gave informed written consent prior to the experiments and were paid 8 €/h as compensation.

### Apparatus

The experiments were performed in a light- and soundproof room. Stimuli were generated using the Psychophysics Toolbox extensions PTB-3 (Brainard, 1997; Kleiner et al., 2007) for MATLAB (MATLAB R2011b; MATLAB. (2011). version 7.13 (R2011b). Natick, Massachusetts: The MathWorks Inc.). They were projected on a large flat screen (120 × 90 cm, 70-cm viewing distance, 81° × 65° visual angle, respectively) using a Christie DS+6K-M projector (running at 1,152-pixel × 864-pixel resolution and 120-Hz refresh rate).

The subjects were seated at a table with the head supported by a chin rest. Eye position was recorded binocularly at 500 Hz using an infrared eye-tracking system (EyeLink II Head Mounted Eye Tracker System; SR Research, Ottawa, Ontario). Only data of the dominant eye, which previously had been determined with a Porta's test (Porta 1953), were analyzed. Before each block of trials, the system was calibrated and validated by matching the current gaze position to a 13-point grid with predefined screen locations. The threshold for a successful calibration was set to a mean deviation of 0.5°.

### Paradigms

**Figure 1.**
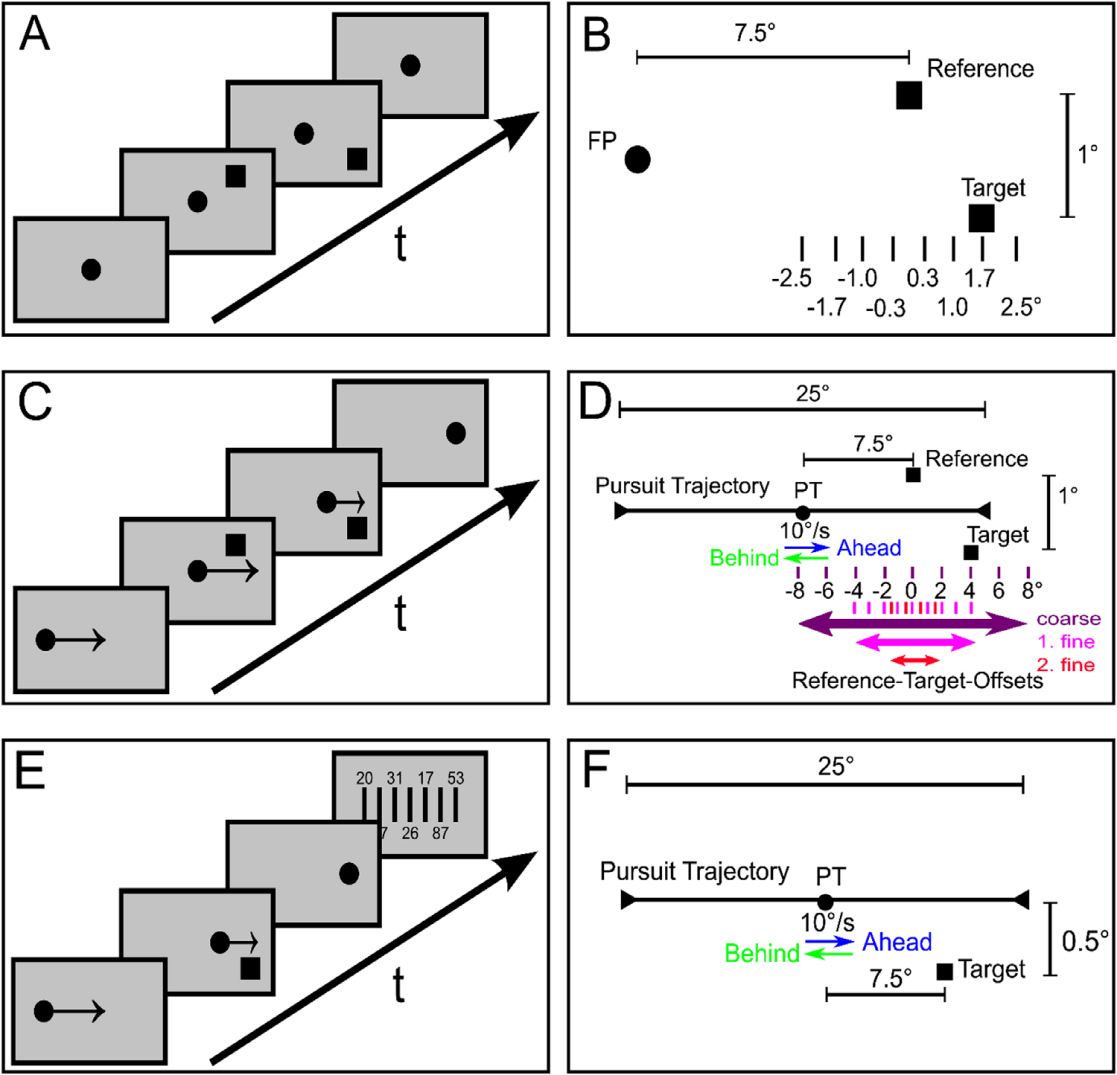
Temporal (left panels) and spatial stimulus configuration (right panels) of the “Fixation Relative” paradigm (A, B), the “Smooth Pursuit Relative” paradigm (C, D), and the “Smooth Pursuit Absolute” paradigm (E, F). All illustrations are exemplary for stimulus presentations in the right hemifield and a positive stimulus onset asynchrony. For stimuli presented in the left hemifield, the reference and target positions were mirrored at the vertical meridian. (A, B) The fixation point (FP) was shown at the center of the screen throughout the trial. The reference stimulus (upper square) was always presented at the same eccentricity of 7.5°. The target stimulus (lower square) was flashed randomized at eight possible positions between ± 2.5° relative to the reference stimulus. (C, D) The pursuit target (PT) moved on a horizontal trajectory with a speed of 10°/s. The position of the pursuit target at the moment when the reference was presented was the same as for the fixation point in A, i.e. the center of the screen. The reference and target stimulus were presented ahead of the pursuit target for rightward pursuit and behind the pursuit target for leftward pursuit. In a first iteration, target stimulus positions were coarsely chosen to determine the general characteristic mislocalization for every subject and condition. Resulting values were used to determine the center around which the target stimulus positions were arranged symmetrically with smaller distances of 1° (1. fine) and finally 0.5° (2. fine). Only data of the two “fine” paradigms were used for further analysis. (E, F) The PT moved on a horizontal trajectory with a speed of 10°/s. Here, only the target stimulus was presented ahead of the pursuit target for rightward pursuit and behind the pursuit target for leftward pursuit at a distance of 7.5° from the center of the screen.

The study consisted of three experiments: a relative localization task during fixation (“Fixation Relative”), a relative localization task during pursuit (“Smooth Pursuit Relative”), and an absolute localization task during pursuit (“Smooth Pursuit Absolute”) (Figure 1). For the relative localization tasks, we adapted the paradigm described by Bocianski et al. (2008) using the method of constant stimuli in a two-alternative forced-choice task. Before starting with the actual experiment, subjects first performed a training session to get used to the experiment and to make sure that they understood the task.

#### Temporal and spatial stimulus configuration

All stimuli were presented on a light gray background (luminance 25.8 cd/m²). The fixation point or pursuit target was a dark dot (luminance 0.2 cd/m², diameter 0.36°), and the localization stimuli consisted of two dark squares (luminance 0.2 cd/m², size 0.36° × 0.36°), an upper square (reference stimulus), and a lower square (target stimulus) with a vertical distance of 1°. The eccentricity of the reference stimulus was held constant at 7.5° either in the left or right visual hemifield.

In the “Fixation Relative” paradigm, one trial lasted 1,750 ms (Figure 1A). Before each trial, a drift correction of the recorded eye position was performed. To this end, the subject had to fixate a small circular ring in the center of the screen and confirm fixation by pressing the space bar. The ring then turned into a filled circle, the fixation point, which started an experimental trial. Subjects were asked to keep fixation on that point throughout the trial. The reference stimulus was presented 1,250 ms after the start of the trial for one frame (about 8 ms) at a fixed position slightly above the horizontal meridian and either 7.5° to the left or to the right of the central fixation point. The target stimulus was flashed for one frame at varying positions of ± 0.3°, ± 1.0°, ± 1.7°, and ± 2.5° (Figure 1B) with respect to the position of the reference stimulus and slightly below the horizontal meridian with three different stimulus onset asynchronies: a “simultaneous” presentation with a SOA of 0 ms and a presentation 200 ms before (“neg. SOA”) or 200 ms after the reference stimulus (“pos. SOA”). At the end of each trial, the subject had to report if the upper or lower stimulus had been perceived more to the right using the up or down key of the keyboard.

The “Smooth Pursuit Relative” paradigm was designed analogous to the one for fixation, but now each trial lasted 2,500 ms (Figure 1C). The initial fixation target (ring) for the drift correction was presented with a 12.5° offset from the center of the screen randomized in either the left or the right hemifield. After pressing the space bar, the ring turned into a filled circle, the pursuit target, and moved with a speed of 10°/s on a horizontal trajectory centripetally. Subjects were asked to track the pursuit target as accurately as possible. After 1,250 ms, when the pursuit target was passing the center of the screen, the reference stimulus was flashed randomly at 7.5° eccentricity, either in the right or left hemifield. This ensured that the reference targets were presented at the same positions on the screen in all “relative” paradigms. Consequently, there were four spatial conditions: “Rightward Ahead” (pursuit target moving to the right, stimuli flashed ahead of the pursuit target), “Leftward Ahead”, “Rightward Behind” (pursuit target moving to the right, stimuli flashed behind the pursuit target), and “Leftward Behind”. The target stimulus was again presented with three SOAs (0 ms, ± 200 ms). In order to determine the location of the target, a pre-experiment (“Smooth Pursuit Relative (coarse)”) was performed for each pursuit condition and subject in order to set the limits for further fine-scaled measurements (Figure 1D). In the coarse mapping, the position of the target was varied with respect to the reference stimulus by 0°, ± 2°, ± 4°, ± 6°, and ± 8°. After the individual offsets of spatial perception due to the ongoing eye movement had been determined, targets were then flashed around these offsets at 0°, ± 1°, ± 2°, ± 3°, and ± 4° in a “Smooth Pursuit Relative (1. fine)” experiment. Using the results from these experiments, we refined the individual localization offsets and the spacing between possible target positions even further in a third iteration (“Smooth Pursuit Relative (2. fine)”). Here target positions were varied by 0°, ± 0.5°, ± 1°, ± 1.5°, and ± 2°. Both the reference and the target stimulus were flashed for one frame during steady-state pursuit. Finally, and similar to the fixation experiment, the subject had to report which of the two stimuli had been perceived more to the right. With this approach, it was possible to cover the entire working range of the psychometric function and get a high resolution of data points around the point of subjective equality (PSE) in a time-efficient way that still allowed for individual differences of spatial perception. For the actual analysis, only data of the two “fine” paradigms were used.

The “Smooth Pursuit Absolute” paradigm was designed analogous to the “Smooth Pursuit Relative” experiment with the same four spatial (Rightward Ahead, Leftward Ahead, Rightward Behind, and Leftward Behind) and three temporal SOAs (0 ms, ± 200 ms) conditions for the presentation of the target stimulus. However, no additional reference stimulus was shown (Figures 1E,F). At the end of each trial, the pursuit target disappeared and a ruler stimulus covering the area between –20° and 20° was shown. The ruler consisted of vertical lines with a distance of 0.8°, respectively. The lines were labeled with numbers between 00 and 99 that were randomly assigned to the vertical lines for each trial to avoid habituation effects. Subjects then had to judge which line corresponded best to the perceived target stimulus position and reported its assigned number using the keyboard. Subjects could correct their input as often as needed and eventually confirm using the enter key. In half of the trials of each condition, the position of the ruler was shifted by 0.4° to increase the resolution by averaging across data from both conditions (i.e., non-shifted and shifted ruler). In each paradigm, all combinations of conditions were presented in a pseudo-randomized order.

### Data processing

Data were collected until there were at least 150 valid trials for each of the 12 pursuit conditions (2*2*3: leftward and rightward pursuit * ahead and behind pursuit * SOA of –200, 0, 200 ms) of the “Smooth Pursuit Relative (fine)” experiments and 50 valid trials for each condition of the “Smooth Pursuit Absolute” experiment.

#### Trial exclusion

Trials were excluded from further analysis if frame drops of the visual stimuli occurred during the trial (< 1% of all trials) or if the eye position deviated more than 2° from the current pursuit target or fixation point up to 50 ms before or after the reference flash. Furthermore, saccades or blinks up to 100 ms before or after stimulus presentation led to omission of the respective trial from further analysis (e.g., Kerzel et al., 2006). Together with the SOA of ± 200 ms, this resulted in a time window of 400 ms for saccade and blink exclusion.

#### Psychometric functions

For every condition and every subject, we computed the ratio of valid trials in which the target stimulus was perceived more eccentric than the reference stimulus. Psychometric functions were fitted to these data using the Psignifit 3.0 toolbox (psignifit.sourceforge.net; cf. Fründ et al., 2011) for MATLAB and a cumulative Gaussian as sigmoid. The respective PSEs were derived as the 50% thresholds between the upper and the lower asymptote. Furthermore, 95% confidence intervals for the respective PSE were provided by the Psignifit 3.0 toolbox and are presented in the Results section. A positive PSE indicates that the target stimulus had to be presented more eccentric than the reference stimulus in order to compensate for less eccentric perception of the target and vice versa.

For analysis of the “Fixation Relative” task, the data for the “right” and the “left” condition were pooled and then the psychometric functions were fitted for each subject and timing condition. For the “Smooth Pursuit Relative” task, data from the two “Smooth Pursuit Relative (fine)” conditions with smaller interstimulus distances, i.e. 1° steps and 0.5° steps, respectively were pooled. For every subject, psychometric functions were fitted for all three timing and four motion conditions (left vs. right; ahead vs. behind). For further analysis, the fitting parameters were averaged across the rightward and leftward conditions.

### Modeling sequential localization during Smooth Pursuit

In order to gain a deeper insight into the underlying mechanism of localization, we modeled the relative localization of two successively presented stimuli under different assumptions, e.g., a shift of the reference frame during SPEMs as compared to fixation. By comparing the model predictions with our behaviorally measured data, we aimed to identify the most suitable reference frame in which this type of localization is performed (see, e.g., Keith et al., 2009, for a comparable approach based on neurophysiological data from the monkey).

#### Retinal versus extraretinal reference frame

**Figure 2.**
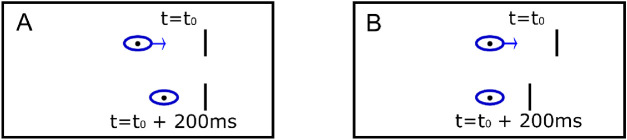
Comparison of the screen-centered (A) and the eye-centered (B) coordinate system for successively presented stimuli (“pos. SOA, Ahead”). Two stimuli are presented successively during an ongoing eye movement at the same position in screen(world) coordinates. In a head-/screen-centered reference frame (A), the eye position shifts, but both stimulus positions are aligned. In an eye-centered reference frame (B), the eye remains at the center of the frame of reference, but the second stimulus is shifted with respect to the first.

We modeled two different scenarios in which relative localization could take place, i.e. a retinal or an extraretinal reference frame. If relative localization was performed in an extraretinal reference frame, there should be no difference in localization between fixation and SPEMs, indicated by equal PSEs. Consequently, if presented at the same location, the reference and the target stimulus would also be perceived at the same location in the SPEM condition, although the eyes would have moved due to the SOA (Figure 2A). However, in a retinal reference frame, the ongoing change of the eye position during SPEMs will induce a characteristic shift to the relative localization of the two successively presented stimuli as compared to steady fixation, indicated by different PSEs. For instance, for a positive SOA of 200 ms, the eyes would have moved 2° in the time interval between the reference and the target stimulus, leading to different relative localizations of the two stimuli (Figure 2B). Therefore, localization during ongoing SPEMs should be a combination of the localization during fixation with a retinal shift with the size corresponding to the SOA.

#### Accounting for target eccentricity

**Figure 3.**
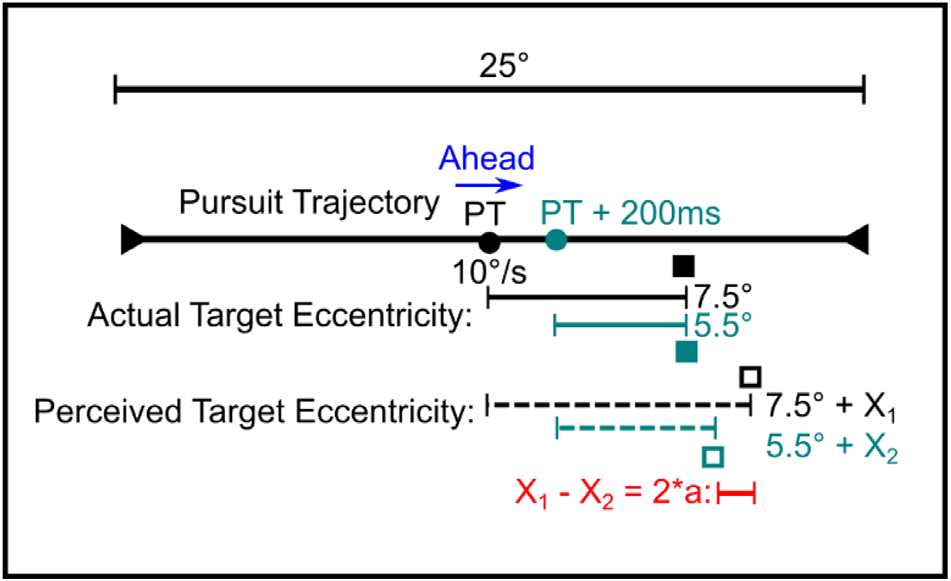
Illustration of the different stimulus eccentricities in the pursuit paradigm. The actual positions of two stimuli presented with no SOA (black filled square) and +200 ms SOA (cyan filled square) can be equal in screen coordinates. Yet, due to the ongoing eye movement toward the target, the retinal eccentricity decreases over time, i.e. it is 2° less eccentric relative to the fovea. The perceived position of the stimulus with no SOA (black empty square) is more eccentric than the position of the stimulus with +200 ms SOA (cyan empty square). To account for this influence of retinal eccentricity on spatial localization, the actual position of the stimulus with +200 ms SOA would have to be shifted by 2*a, where “a” is the slope of the linear regression of the individual absolute mislocalizations (X_1_, X_2_, etc.) as a function of retinal eccentricity. Therefore, “2*a” indicates the eccentricity effect on localization caused by a 2° shift of the retinal stimulus position.

Due to the ongoing eye movement in the pursuit paradigms, the different presentation times of the two flashed stimuli led to different retinal eccentricities at which each stimulus was presented (Figure 3). To determine a potential influence of stimulus eccentricity on the relative localization judgment in the “Smooth Pursuit Relative” conditions, we performed a linear regression analysis as follows: The difference between perceived and actual target position, i.e. the magnitude of mislocalization was expressed as a function of the retinal flash position for each subject, using the pooled data of the “Smooth Pursuit Absolute” experiment.

The slope “a” of the linear regression function represents an eccentricity effect caused by a 1° shift of the presented target stimulus position in retinal coordinates. With this, the shift of the PSE required to compensate for an eccentricity effect, which is caused by the 2° shift of the eye position due to the SOA, can be derived for the different SOA conditions (see Table 1).

**Table 1.** Shifts of PSEs necessary to compensate the eccentricity effect.

	Ahead	Behind
Pos. SOA	+2 × a_ahead_	–2 × a_behind_
Neg. SOA	–2 × a_ahead_	+2 × a_behind_

In order to account for a potential eccentricity effect, we extended the two models based on different reference frames with a computed PSE shift that was obtained by the abovementioned regression analysis. This resulted in a total of four different models that we compared with our experimental data: (a) a head-/screen-centered coordinate frame without an eccentricity effect, (b) an eye-centered reference frame without an eccentricity effect, (c) a head-/screen-centered reference frame with an eccentricity effect, and (d) an eye-centered reference frame with an eccentricity effect.

### Statistical tests

In order to effectively keep Type I errors low, a sequential Bonferroni correction for multiple tests has been applied to the results (Holm, 1979). To this end, all tests have to be calculated first. The results are then ordered from the smallest to the largest *p* value. The test with the lowest *p* value is tested first with a Bonferroni correction factor representing the total number of tests performed. The test with the second lowest *p* value is then tested with a Bonferroni correction factor involving one test less and so on for the remaining tests. This approach is less conservative than the standard Bonferroni correction and thus more powerful in detecting truly significant effects (Abdi, 2010). In the Results section, we will provide *p* values that were significant after applying Holm's correction for each type of test. Furthermore, we calculated Cohen's *d_z_* from the *t* value of the statistical tests, divided by the square root of the number of participants as a measure of effect size (Lakens, 2013).

## Results

### Relative localization

**Figure 4.**
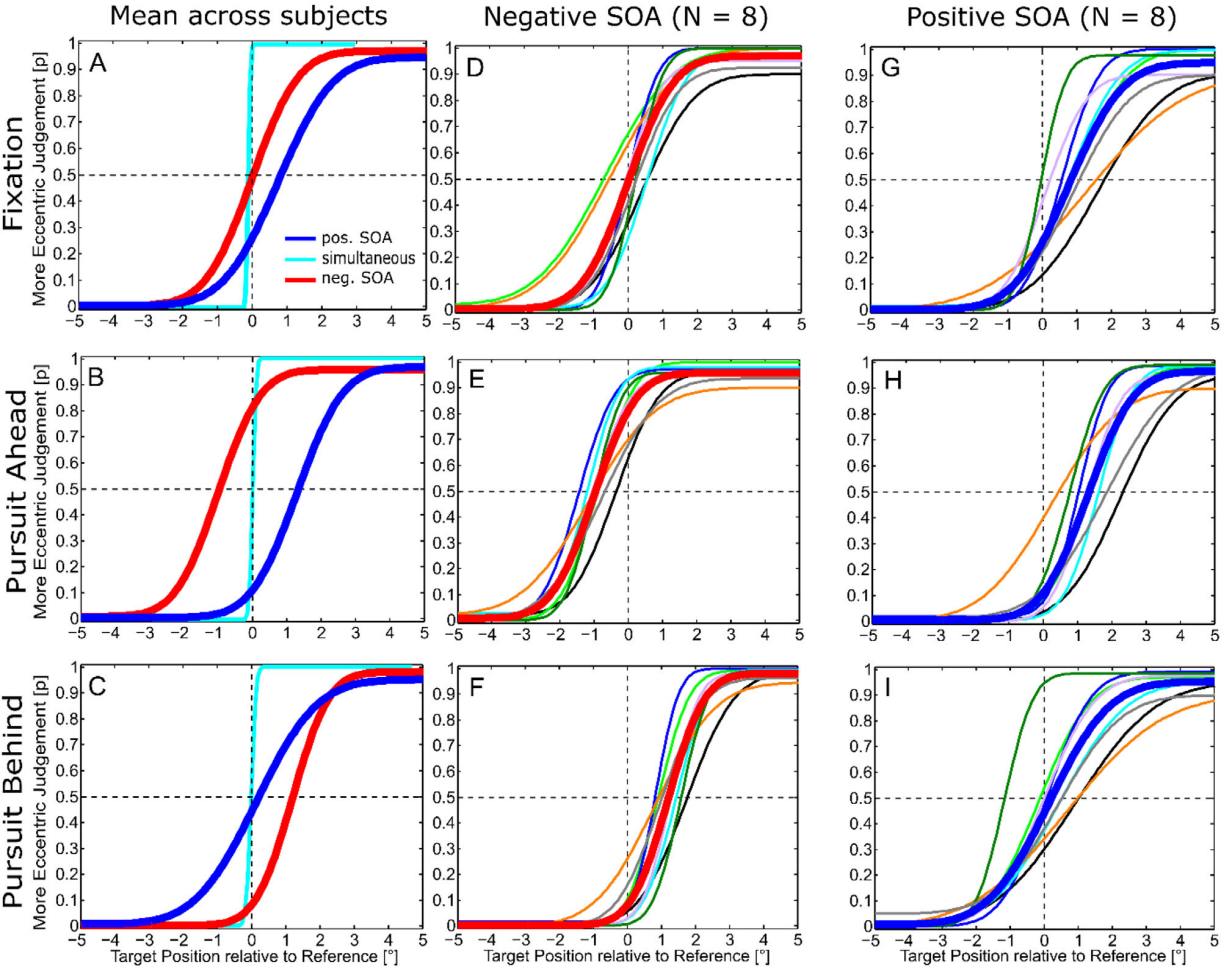
Relative localization performance averaged across all subjects (A–C) and all subjects individually (D–I). The relative frequency of a more eccentric judgment of the target stimulus is plotted against the relative target position with respect to the reference stimulus in screen coordinates (positive values indicate a more eccentric target position). (A–C) Relative localization performance averaged across all subject in the “Fixation Relative” paradigm (A) and the “Smooth Pursuit Relative (fine)” paradigm with targets ahead (B) or behind (C) of the pursuit target. The three different SOAs between the reference and the target stimulus are color-coded. A positive shift of the point of subjective equality indicates that the target had to be presented more eccentric in order to be perceived at the same position as the reference, i.e. the target was localized more toward the fovea and vice versa. (D–I) Relative localization performance individually for all eight subjects. The Psychometric Functions are color-coded for the respective subjects; the average function over all subjects is colored in bold red for negative SOAs and bold blue for positive SOAs. Panels D through F show results for the different motion conditions when the target was presented with a SOA of –200 ms; G through I are analogous for a SOA of +200 ms. Data of the “Fixation” paradigm (D, G) were pooled across presentation in the left and right hemifield. During the “Pursuit” paradigms, we distinguished between the “Ahead” (eyes moving toward the localization stimuli flashed; E, H) and the “Behind” (eyes moving away from the localization stimuli flashed; F, I) condition. Data of the “Leftward” and “Rightward” condition were averaged. A shift of the psychometric function toward positive values indicates that the target was perceived more toward the fovea and vice versa.

We analyzed the relative localization performance of two briefly flashed stimuli during fixation and smooth pursuit eye movements. Figure 4 shows the average performance across all subject for the different conditions (A–C) and individually for all eight subjects (D-I).

In the “simultaneous” conditions (data shown only for the average across subjects in Figures 4A–C), the average PSE of all subjects was close to zero for all three types of eye movements (Fixation: –0.02° ± 0.17°; Pursuit Ahead: 0.07° ± 0.07°; Pursuit Behind: 0.09° ± 0.06°), indicating that all subjects were able to correctly report which of the two stimuli was presented more eccentric.

When the target was presented with a negative SOA, the average PSE value was 0° ± 0.42° in the “Fixation Relative” paradigm (Figure 4D). In the “Ahead” condition of the “Smooth Pursuit Relative” paradigm (Figure 4E), the average PSE was shifted toward lower eccentricities (PSE = –1.0° ± 0.32°), that is, subjects reported the target stimulus more eccentric in comparison to the reference stimulus. In the “Behind” condition, the PSE was shifted toward larger eccentricities (PSE = +1.19° ± 0.36°, Figure 4F), indicating that the subjects perceived the target closer to the fovea as compared to the reference when flashed at the same position. The shift of the PSE was significantly different in both pursuit conditions, as compared to the fixation condition (“Ahead” vs. Fixation: *t*(7) = –5.91, *p* < 0.001, *d_z_* = 2.09, two-tailed paired-sample *t* test; “Behind” vs. Fixation: *t*(7) = 11.56, *p* < 0.001, *d_z_* = 4.09, two-tailed paired-sample *t* test). In addition, the difference of the PSEs in the “Ahead” compared to the “Behind” condition was statistically significant (*t*(7) = –18.63, *p* < 0.001, *d_z_* = 6.59, two-tailed paired-sample *t* test).

When the target stimulus was presented with a positive SOA in the “Fixation Relative” paradigm (Figure 4G), it was mislocalized closer to the fovea with respect to the reference stimulus. The average PSE value was +0.75° ± 0.55°, which was significantly larger than during the negative SOA condition (*t*(7) = 3.00, *p* = 0.01, *d_z_* = 1.06, one-tailed one-sample *t* test). Compared to fixation, the mislocalization increased when the eyes were moving toward the flashed stimuli (Figure 4H; PSE = +1.3° ± 0.63°) and decreased for pursuit away from the flashes (Figure 4I; PSE = +0.14° ± 0.32°). This time, for a positive SOA, the shift of the PSE was significantly different between “Pursuit Behind” and the fixation condition (*t*(7) = –5.28, *p* = 0.001, *d_z_* = 1.87, two-tailed paired-sample *t* test) and showed a trend when comparing “Pursuit Ahead” and fixation (*t*(7) = 2.29, *p* = 0.055, *d_z_* = 0.81, two-tailed paired-sample *t* test). Furthermore, the difference between PSE values for the “Ahead” as compared to the “Behind” condition was statistically significant (*t*(7) = 4.67, *p* = 0.002, *d_z_* = 1.65, two-tailed paired-sample *t* test). Similar to the fixation paradigm, during “Pursuit Ahead,” the average PSE value was significantly higher during the “pos. SOA” condition as compared to the “neg. SOA” (*t*(7) = 13.57, *p* < 0.001, *d_z_* = 4.80, two-tailed paired-sample *t* test). On the other hand, in the “Pursuit Behind” condition, the average PSE value was significantly smaller for positive SOAs as compared to negative SOAs (*t*(7) = –3.90, *p* = 0.006, *d_z_* = 1.38, two-tailed paired-sample *t* test).

### Absolute localization and eccentricity effect

**Figure 5.**
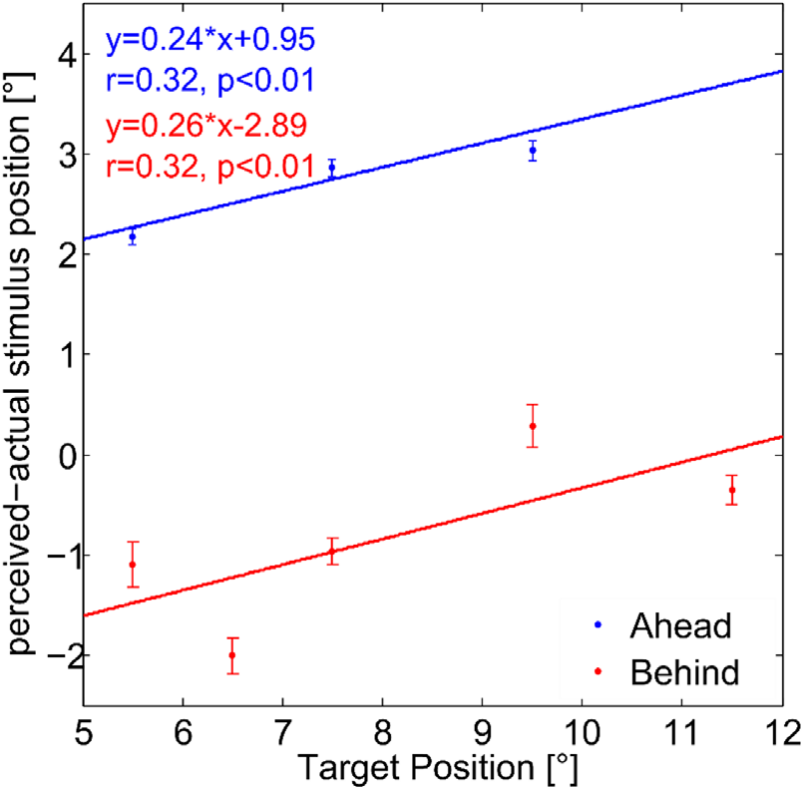
Mislocalization as a function of absolute retinal stimulus eccentricity in eye coordinates in the “Ahead” (blue) and “Behind” condition (red) for one representative subject. The difference of the perceived position (as reported by using the ruler) and the actual target stimulus position (as presented on the screen) is plotted against the target position in eye coordinates. Larger values on the *y*-axis indicate a more eccentrically perceived position as compared to the actual position, whereas larger values on the *x*-axis indicate more eccentrically actual positions. For each target position, the mean mislocalization and the corresponding standard error are shown. Linear regression functions were fitted and plotted for both the “Ahead” and the “Behind” conditions. The slopes of the functions indicate the effect of the eccentricity on the mislocalization. For this subject, in the “Behind” condition, five target positions were tested to determine the influence of eccentricity in greater detail.

When the target and the reference stimulus were presented successively during the ongoing pursuit eye movement, the retinal position of the first and the second flash varied as compared to a simultaneous presentation of both stimuli. With a pursuit target speed of 10°/s and a gain close to 1.0, an SOA of ± 200 ms should lead to a difference in retinal position of approximately 2°. Indeed, the average eye velocity as determined by the eye tracker closely matched the pursuit target speed in the relevant time window from 100 ms before until 100 ms after flash presentation (gain: 1.02 ± 0.03). To examine if stimulus eccentricity had an effect on the localization performance, the “Smooth Pursuit Absolute” task was performed. Data of one representative subject are shown in Figure 5. A linear regression analysis was applied to the data of the “Ahead” and “Behind” condition, respectively.

When presented with a retinal eccentricity of 7.5° in the “Ahead” condition, the flash was on average mislocalized by about 2.75° in the direction of the ongoing pursuit eye movement. This is equivalent to a more outer judgment with respect to the fovea or the center of the screen. A positive slope of the linear fit implies that the more eccentric a stimulus is, the more eccentric it is mislocalized. Furthermore, the value of the slope describes the magnitude of the absolute mislocalization as a function of retinal eccentricity. Consequently, if the target was presented in the “Ahead” condition with a positive SOA of 200 ms (eye position shifted by 2° within the interstimulus interval), the perceptual shift was on average 0.48° smaller than for the more eccentric reference stimulus. Accordingly, to compensate for this eccentricity effect, the PSE has to be shifted by +0.48° with respect to a fixation condition.

When presented in the “Behind” condition, the flash was also mislocalized in the direction of pursuit but with a much smaller absolute shift as compared to the “Ahead” condition (*t*(7) = 2.95, *p* = 0.021, *d_z_* = 1.03, two-tailed paired-sample *t* test with averaged data of all subjects). In addition, due to the pursuit direction, this perceptual shift was not away from the fovea or the center of the screen but toward it (mean perceived location = 6.55° when presented at 7.5°). For more eccentric flash positions, this perceptual shift toward the fovea became smaller. Just as for the “Ahead” condition, in the “Behind” condition, the slope of the linear regression curve describes the magnitude of the absolute mislocalization as a function of retinal eccentricity. In this case, a slope of 0.26 indicates that the target presented in the “Behind” condition was perceived on average 0.52° more eccentric than the reference stimulus when presented with a positive SOA of 200 ms, resulting in an eye position shift of 2° within that time. To compensate for this potential eccentricity effect, the PSE has to be shifted by –0.52° with respect to the PSE of the “Pos. SOA” condition in the “Fixation Relative” paradigm. Likewise, the eccentricity effect on the PSE values in the “Ahead” and in the “Behind” condition was determined for each subject (Table 2).

**Table 2.** Required PSE shifts to compensate for the eccentricity effect for all subjects in the Ahead and Behind conditions of the smooth pursuit task.

Subject	Ahead	Behind
1	+0.99°	–0.55°
2	+0.59°	–0.81°
3	+0.48°	–0.52°
4	+2.40°	–0.43°
5	+0.74°	–0.73°
6	+0.86°	–0.75°
7	+0.40°	–1.63°
8	+0.31°	–0.54°
Mean	+0.85° ± 0.67°	–0.74° ± 0.38°

### Comparison of models for the mislocalization during pursuit

**Figure 6.**
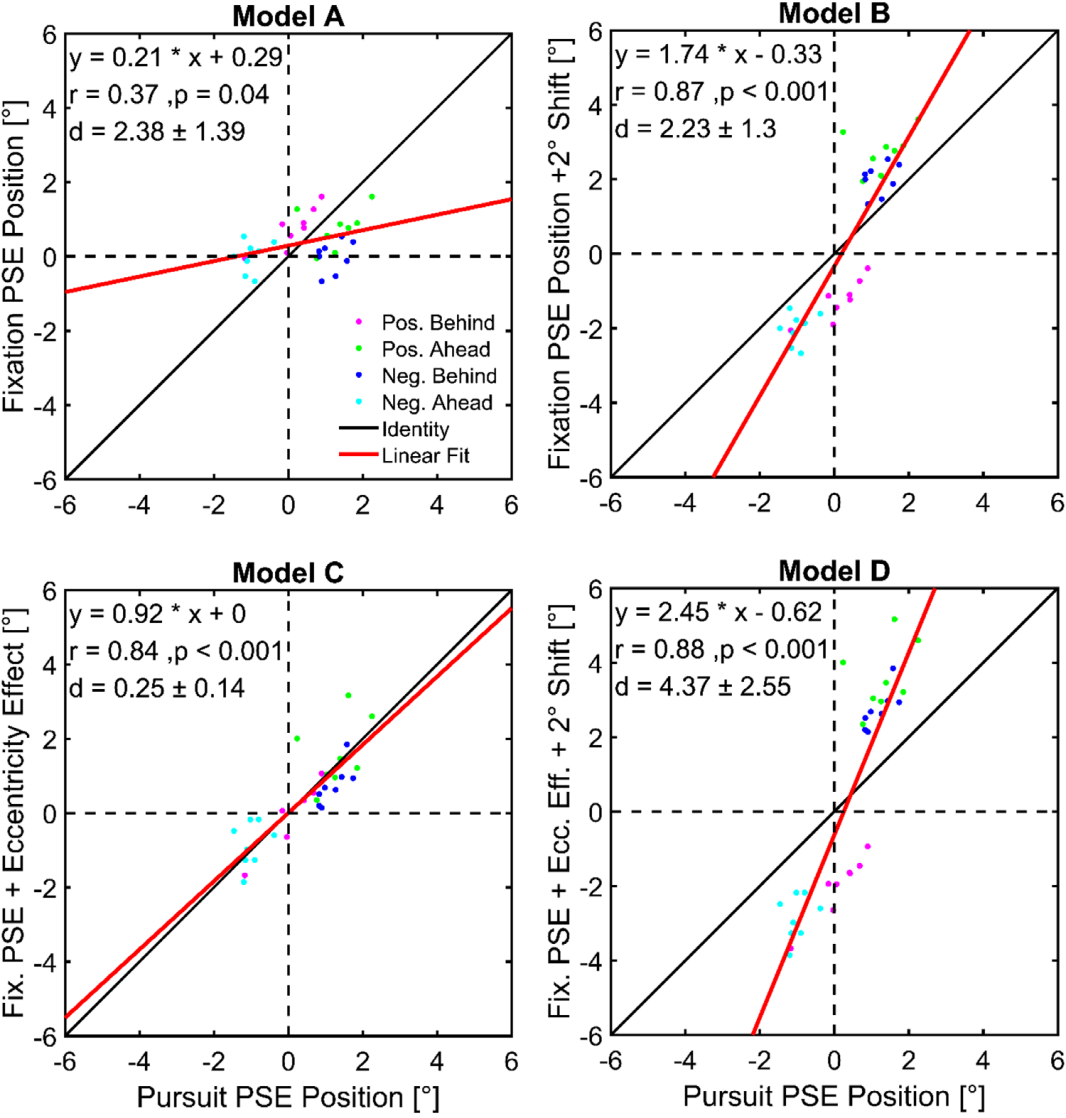
Comparison of different model predictions with the behaviorally measured relative mislocalization during pursuit eye movements for the combination of the different SOAs and “Ahead”/“Behind” conditions. The PSE values as predicted by each model are plotted against the respective PSE values measured in the “Smooth Pursuit Relative” paradigm. The different SOAs and “Ahead”/“Behind” conditions are color-coded. Each data point represents data from one subject. Linear regression curves were fitted to the data and compared to identity. In Model A, we compared the mislocalization of the “Fixation” paradigm to the respective values during ongoing pursuit eye movements. In Model B, we added a 2° shift due to the changing eye position within the interstimulus interval; Models C and D were analogous while also considering an effect of stimulus eccentricity.

In order to explain the relative mislocalization effect during smooth pursuit, we modeled four different spatial encodings to predict the experimental findings. With Model A, we hypothesized that perception is not altered by the pursuit eye movement, resulting in indistinguishable PSEs in the “Fixation Relative” and the “Smooth Pursuit Relative” paradigm. In Model B, we added a 2° shift to the fixational PSEs, assuming that mislocalization during ongoing pursuit can be explained by a combination of the mislocalization during fixation and a retinal shift due to the changing eye position within the interstimulus interval. Furthermore, with Models C and D, we considered an eccentricity effect, as determined for each subject with the “Smooth Pursuit Absolute” paradigm. Accordingly, in Model C, we combined this effect with the fixation data (analogous to A), while in Model D, we also added a 2° shift equivalent to B. We calculated the PSE values as predicted by each model for each subject and condition and analyzed the model performance as a function of the respective PSE values measured in the “Smooth Pursuit Relative” paradigm (Figure 6). The closer the data points and their linear fits approximate the identity line, the better the match between model and measured data. Accordingly, an ideal model would lead to a slope of the fit close to +1.0. Furthermore, the mean pointwise distance “d” of the data points to the identity line would be close to zero. Overall, Model C predicted the experimental data best. There was a high correlation of the data points (*r* = 0.84, Pearson correlation) and the slope of the linear regression with a value of 0.92 was close to 1. Furthermore, the pointwise distance of the data points from identity was very small (d = 0.25 ± 0.14), indicating a high similarity of data and model. In comparison, Model A underestimated the measured data with a slope of 0.21. Consequently, the pointwise distance from identity (d = 2.38 ± 1.39) was larger than for Model C. Likewise, Models B and D did not predict the pursuit PSE positions very well. These two models overestimated the data with slopes of 1.74 for Model B and 2.45 for Model D. This led to considerable deviations from the identity line with an average pointwise distance of d = 2.23 ± 1.3 for Model B and d = 4.37 ± 2.55 for Model D.

## Discussion

### Relative localization

The goal of this study was to examine if and how ongoing pursuit eye movements affect the relative localization performance observed during fixation. Furthermore, this approach allows identifying the reference frame in which relative localization might take place. Therefore, we analyzed PSE positions for different pursuit conditions and compared these to the values in the respective fixation conditions. First, the results in the fixation paradigm were in accordance with the findings of Bocianski et al. (2008). The larger mislocalization effect for the “pos. SOA” condition in the current study might have been a consequence of small modifications to the paradigm, i.e. using a SOA of 200 ms instead of 100 ms, decreasing the vertical distance between both flashes from 1.4° to 1° and presenting the flashes further in the periphery. Second, in the “pos. SOA” and the “neg. SOA” conditions of the “Smooth Pursuit Relative” paradigm, the PSE values differed significantly between the “Ahead” and the “Behind” conditions. Furthermore, the PSE values in both conditions differed significantly from the respective values in the fixation condition. This indicates that the pursuit eye movement had influenced the relative localization performance, since the eyes moved on during the interstimulus interval. More specifically, a systematic pattern concerning the directions of the PSE shifts could be observed: The average PSE shifted more eccentric as compared to the mean PSE in the fixation paradigm for both the “pos. SOA, Ahead” and the “neg. SOA, Behind” condition (Case (i)). The shift was reversed for the “neg. SOA, Ahead” and the “pos. SOA, Behind” condition (Case (ii)). The main difference between these two cases was that in Case (i), the average target position was closer to the fovea than the reference stimulus, whereas in Case (ii), it was further away due to the ongoing eye movement during the SOA period. The reference stimulus was always shown at the same eccentricity when the pursuit target was passing the center of the screen. Hence, the distance between the fovea and the average target stimulus position affected the relative mislocalization of the two flashes.

### Absolute localization during SPEMs and effects on the relative localization

In order to correctly quantify the effect of smooth pursuit eye movements on the relative localization performance, we measured the absolute localization of a single flash at different retinal eccentricities. This allowed us to determine if the deviations of the PSEs during pursuit as compared to fixation originated from the different absolute mislocalization of the single flash due to different eccentricities during ongoing SPEMs. For both the “Ahead” and the “Behind” conditions, the target was perceptually shifted in the direction of the pursuit with a stronger mislocalization in the “Ahead” condition. In addition, we found a significant effect of the retinal target eccentricity on the absolute localization performance. For presentation in the “Ahead” condition, the mislocalization increased with increasing retinal eccentricity, whereas the mislocalization in the pursuit direction decreased, when the target was presented further away behind the pursuit target. This asymmetric mislocalization pattern is a common finding in human behavioral studies (e.g., van Beers et al. 2001; Königs & Bremmer, 2010). A recent study by Dowiasch et al. (2016) has found a neural correlate of this characteristic mislocalization pattern in the parietal cortex of the animal model of human sensorimotor processing, i.e. VIP area in macaque monkeys. Their model combined an eye position signal, which could be decoded from neuronal discharges and which was slightly ahead of the actual eye position, with a representation of perceptual space distorted by attention. For the latter, the distribution of attention during pursuit, mapped by Khan et al. (2010), was used. By mapping response latencies to visual stimuli presented around the pursuit target, these authors showed that attention is allocated broadly ahead of the pursuit target with a peak of attention at about 4° ahead of the pursuit target. With the assumption that the location with the most focused attention shows the smallest mislocalization, this effect would predict the asymmetric mislocalization between the “Ahead” and “Behind” conditions.

It has been shown that mislocalization can be strongly influenced by spatial attention (Bocianski et al., 2010). In both the fixation and the pursuit paradigms, stimuli could be presented to the left or the right of the fovea. Accordingly, we assume that spatial attention was broadly allocated in both cases. Yet, it has been shown before that during smooth pursuit, spatial attention is slightly biased toward a region in front of the pursuit target (Khan et al., 2010; Dowiasch et al., 2016; Chen et al., 2017). Accordingly, we assume that this pursuit-induced reallocation of the focus of attention away from the pursuit target (as compared to the fixation target in the fixation condition) might have induced an asymmetry in (mis)localization, which, however, most likely was small.

In the relative mislocalization task, the mean retinal eccentricity of the two flashes differed by approximately 2° due to the ongoing eye movement during the interstimulus interval. Therefore, the deviating absolute mislocalization of each of the two flashes could cause the changes in the relative mislocalization as compared to the fixation task. Yet, the shift of the retinal image during the interstimulus interval could also directly affect the localization performance. Therefore, we compared different models to evaluate which frame of reference was more appropriate to predict the behaviorally measured data.

### Evaluation of the models

To explain the measured deviations of the PSE values during fixation and pursuit, we developed four models and compared their predicted PSEs with the behaviorally measured values in the different pursuit conditions. Furthermore, each model was related to a frame of reference in which the relative mislocalization was observed. Two of the models (B and D) were based on an eye-centered coordinate system, while the other two (A and C) were based on a head- or screen-centered frame of reference. The design of the models supposed that the eye position and thus the retinocentric reference frame shifted by 2° during the interstimulus interval (SOA: ± 200 ms, pursuit target speed: 10°/s). Since the gain of the steady-state pursuit of our subjects was close to 1.0 as expected for this target speed (Barnes, 2008; Lisberger et al., 1981; Konen et al., 2005), this is a sufficiently accurate approximation.

According to Models B and D (i.e., localization in an eye-centered reference frame), the movement of the eyes would lead to a shift of the PSEs by the same amount. To compensate for this, the PSEs in the pursuit conditions had to be shifted by 2° as compared to the PSEs for fixation. When presented with a “pos. SOA” in the “Ahead” condition, the PSEs were shifted toward higher eccentricities by +2° compared to the respective values in the fixation paradigm, whereas they were shifted toward lower eccentricities for presentation in the “Behind” condition. The directions of the shifts predicted by Models B and D were in line with the behaviorally measured data in this study. However, the magnitude of the observed shifts was significantly smaller than predicted by the models. Therefore, it is unlikely that the different PSEs can be explained by localization in a retinocentric coordinate system. This conclusion is supported by a study from Cai et al. (1997) showing that the perceived alignment of three dots was systematically altered, when presented briefly before saccade onset. The authors concluded that the relative position perception of perisaccadic stimuli did not exclusively depend on the retinal information but was influenced by the eye movement.

Another hypothesis is that the relative localization judgment is made after the information about the stimulus positions has been transformed from an eye-centered to a head-centered (or even body- or world-centered) coordinate system (e.g., Zipser & Andersen, 1988; Bremmer et al., 1998). Such reference frame transformations at the single cell level have been shown at later stages in the processing of visual information in monkeys, i.e. VIP area; Duhamel et al., 1997; Schlack et al., 2005. If the ongoing pursuit in general and the shift of the eye position during the interstimulus interval in particular would be completely compensated for by this transformation, the visual system would use veridical information about each flash's position in screen coordinates for the relative localization. Thus, the PSEs in the pursuit conditions would equal the respective ones in the fixation task without any additional effects (Model A). However, our results showed that the values differed significantly, with systematic shifts of the PSEs depending on the targets' retinal eccentricity. This shows that the eye movement still had an effect on the relative localization, even if the spatial information was transformed into another coordinate system. Furthermore, it indicates that the eccentricity effect, as observed in the absolute localization task, also needs to be considered for the relative localization. Our Model C, employing a linear combination of the PSEs in the fixation task with shifts to compensate for the eccentricity effect, predicted the behaviorally measured pursuit data the best. In all conditions, the mean deviation between the predicted and the behaviorally measured PSEs was the smallest compared to the other models. Thus, our results suggest that the relative localization judgment occurs at a stage of the visual processing, where spatial information is available in nonretinal frames of reference. This could be an early stage, employing a nonretinocentric encoding via an implicit, i.e. population code (e.g., Bremmer et al., 1998; Boussaoud & Bremmer, 1999). Or it could be based on an explicit encoding at the single cell level, as found in the monkey VIP area (Duhamel et al., 1997). Within this transformation, the representation of each stimulus had been shifted in accordance with its eccentricity, when presented during ongoing SPEMs. Yet, it might be possible that the relative position information had been encoded neither in an eye-centered nor in a head-centered frame of reference but in an intermediate reference frame as also found in the VIP area (Duhamel et al., 1997). A model using only a 1° shift during the interstimulus interval without considering the eccentricity effect was also able to predict the experimentally measured PSEs in three of the four examined pursuit conditions (data not shown). Hence, this model and Model C performed equally well. Nevertheless, both models suggest that the relative localization judgment relies on nonretinocentric information and, therefore, is likely to be performed at a stage of the visual processing in which retinal and nonretinal information is available. Importantly, studies suggest that shorter SOAs can lead to a localization in a retinal reference frame (Brenner & Cornelissen, 2000). In a follow-up study, these authors showed that during saccades, relative localization occurred in a nonretinocentric reference frame for SOAs longer than 200 ms but in a retinocentric reference frame for smaller SOAs (Brenner et al., 2005). These findings were backed up by behavioral studies (Zimmermann et al., 2013) and neurophysiological studies in two gaze control centers of the macaque monkey, that is, the superior colliculus (Sadeh et al., 2020) or frontal eye field (Sajad et al., 2016), which suggest that building nonretinocentric spatial representations requires time on the order of a couple of hundred milliseconds. Taken together, these studies might imply that also in the current experimental approach, mislocalization could have occurred in an eye-centered rather than in a world-centered reference frame, if SOAs would had been shorter. Further experiments, however, are necessary to test this exciting hypothesis. In principle, nonretinal localization can happen already at the level of primary visual cortex (Trotter & Celebrini, 1999; Morris & Krekelberg, 2019), but we consider it more likely to happen in downstream areas of extrastriate or parietal cortex (Duhamel et al., 1997; Schlack et al., 2005). Additional experiments varying head and body positions during localization, however, would be required to differentiate between head-, screen-, body-, and world-centered reference frames.
